# Regional Biophysics Conference 2012: an anniversary and a milestone for applications of biophysics in medicine

**DOI:** 10.3325/cmj.2012.53.515

**Published:** 2012-12

**Authors:** Pavle Andjus

**Affiliations:** Faculty of Biology, Belgrade, Serbia

Dedicated to Greta Pifat-Mrzljak (1939-2009) and Bogdan Beleslin (1935-2002)

## The beginnings of Regional Biophysics Conference

This issue of the *Croatian Medical Journal* (CMJ) presents a series of articles from the Regional Biophysics Conference (RBC) organized in Kladovo, Serbia on September 3-7, 2012. This was the fifth conference in the RBC series, a small but notable anniversary. In the opening speech, Dr Marjeta Šentjurc, the veteran biophysicist from the Jožef Štefan Institute, Ljubljana, Slovenia, focused on the development of the RBC initiative starting from the first meeting in Zreče, Slovenia in 2005. She pointed out that this initiative was a continuation of and an upgrade to the previous Yugoslav Biophysical Society (YBS) meetings, which were traditionally organized each year for more than 40 years – since November 1970. In fact, the initiative for establishing an organization that would gather researchers from the field of biophysics was announced already in 1966 at the Board of the Federal Council for the Coordination of Scientific research, which allocated some funds to the Institute of Physics of the School of Natural Sciences University of Belgrade, Serbia for this purpose. Director of the Institute, Prof. Aleksandar Milojević, PhD and Head of the Biophysics Department at the Institute of Biology in Zagreb, Croatia, Siniša Maričić, PhD, organized a meeting of 26 scientists from the field of biophysics on June 26, 1970 in Zagreb. At the meeting, the Board of the Initiative for Biophysics was elected with the members: B. Beleslin, J. Herak, I. Pavlič, Č. Radenović, and S. Svetina. Their role was to organize the first Yugoslav Scientific Meeting on Biophysics in Krapinske Toplice, Croatia in November 1970. At the meeting, the YBS was founded, the aim of which was to organize further regular meetings and establish the post-graduate school of biophysics. The presentations from the meeting were published in the international journal *Periodicum Biologorum* printed in Croatia. Since then, the meetings were organized regularly each year at venues in Croatia, Slovenia, Serbia, and later in Bosnia and Herzegovina. The last YBS meeting took place in Rogaška Slatina in Slovenia. The meetings have always had a very strong biomedical component and facilitated network-building in the region. Also, besides scientists from the former Yugoslavia, the meeting gathered a considerable number of scientists from Italy, Austria, and Hungary, so it marked a beginning of many collaborations, some of which still last and include a great number of younger scientists. In this respect, these activities heralded today’s trends of multidisciplinarity in research sustained through networking and opening up more opportunities for young researchers. Moreover, stress has always been placed on applicability of research, primarily in biomedicine.

In the following years, many local seminars, round table discussions, and summer schools on the topic of biophysics were organized in Yugoslavia. Among them was the internationally acclaimed International summer school “Supramolecular Structure and Function,” founded and organized by Prof. Greta Pifat-Mrzljak from the Rudjer Bošković Institute, Zagreb, Croatia, which has continued to be organized regularly, even after her death in 2009. In addition, in 1977 YBS was asked by the Federal Agency for International Cooperation in Science to coordinate the activities in the field of biophysics with respect to cooperation with the COMECON (Council for Mutual Economic Assistance) – a kind of Eastern Bloc Organisation for Economic Co-operation and Development (Dr Čedomir Radenović was appointed YBS representative).

All these activities stopped in 1991 with the breakup of Yugoslavia. However, the need to meet colleagues from different areas of biophysics, to exchange experiences and establish collaboration, remained. In September 2002, Slovene scientists in the field of life sciences, as Dr Šentjurc reminded in her opening speech, together with their Italian colleagues organized a joint Italian-Slovenian Workshop in Biochemistry and Biophysics in Trento, Italy. This meeting gave birth to the idea of organizing regional biophysics conferences for experts from the neighboring countries. Thus, the first RBC meeting was organized in 2005 in Zreče by the Slovenian Biophysical Society in cooperation with Biophysical Societies of Austria, Croatia, Hungary, and Italy, and further meetings were agreed to be held regularly each two years in countries of the region. Later the group was broadened to include Serbia and Slovakia. The meetings were held in Balatonfüred, Hungary (2007); Linz, Austria (2009); Primošten, Croatia (2010); and finally Kladovo, Serbia (2012).

As Dr Šentjurc pointed out in her opening speech – the RBC series facilitates the trans-border collaboration of groups with complementary resources allowing for a more economical near-neighbor (as compared to distant centers) exchange of know-how and expert knowledge (1). The 5th conference thus established RBC initiative as a sustainable long-term network.

## Advanced regional trends in Medical Biophysics

An entire section of the meeting, as well as parts of other sections, were devoted to Medical Biophysics. Thus, modern regional trends of employing biophysics in health were well represented. The contributions in this field could be mainly divided in two categories: a) development of techniques for therapy and diagnostics and b) modeling of biomedical phenomena.

Regarding biophysical techniques and approaches in medicine, one of the topics discussed at the conference was a diverse set of particular applications of the advanced use of collimated light sources. Branislav Jelenković from the Institute of Physics in Belgrade presented nonlinear optical microscopes using femtosecond broad band laser pulses for multi-photon excitation and generation of second and third harmonics, which allow for increased depth and unparalleled three dimensional localization of tissues and cells in vivo. In addition to this, 3-D digital holographic microscopy, integrated with numerical processing, enables unique quantitative information for cellular identification. Finally, optical tweezers can provide a unique and flexible laser tool for cell manipulation. In fact, as presented by Lorand Kelemen from the Institute of Biophysics in Szeged, optically driven microtools built by two-photon polymerization can be trapped by optical tweezers providing them with precise movement and position within the cell. The advanced use of light is also apparent in the therapy of diseases such as cancer. Thus, the group of Pavol Miskovsky from the Department of Biophysics in Košice explored a biophysical technique to deliver a photosensitive cytostatic to the targeted tumor tissue by means of low-density lipoprotein carriers. These nano-sensors can be conjugated to metalized nano-beads that allow optical trapping and scanning with nanometric accuracy by surface-enhanced Raman scattering.

An interesting novel approach to the diagnostics of cancer with Raman spectroscopy was also presented by Aleksandra Pavićević and the multidisciplinary team of physical chemists and physicians from the University of Belgrade (2). Namely, micro-Raman spectroscopy combined with neural network algorithm software was presented as a combined tool for the classification of a number of different types of cancers. In many hospitals, particularly in the field of radiology, biophysicists are members of multidisciplinary clinical teams. One example of such collaboration is the work of Božidar Casar of the Institute of Oncology in Ljubljana, who demonstrated how Monte Carlo algorithms may be used for the calculation of accurate dosage in stereotactic radiosurgery of intracranial lesions.

Biophysics is also helpful in modeling of medical phenomena, ie, designing better markers and calculating the best therapeutic approaches (3). Such applications were presented by Aleš Fajmut from the University of Maribor, Nina Bizjak from Jožef Štefan Institute in Ljubljana, Biljana Jakovljević from National Cancer Research Institute in Belgrade, and Ivana Kantardžić from University of Novi Sad. Aleš Fajmut presented the perspective of modeling of airway smooth muscle contraction in normal and pathological conditions that could explain the cellular signaling pathways in aspirin-induced asthma, as well as the therapeutic applications of beta-agonists or rho-kinase inhibitors. Nina Bizjak demonstrated a simple stroboscopic microscope technique for the analysis of the blood clot model that could allow for the prediction of reliable thrombolitic therapy. Mathematical modeling can also contribute to the calculation of the apparent diffusion coefficients with internal membrane barriers, thus supporting the prediction of tumor size with diffusion weighted MRI (presentation by B. Jakovljević). Biomechanical modeling presented by I. Kantardžić can also predict and/or create conditions for the whole tooth design in virtual reality. Thus, by additional simulation of different cavities the 3D tooth model could biomechanically predict the most appropriate design for further clinical research (4).

A particular contribution of modern biophysics is in the rapidly growing field of tissue engineering and biomaterial design for better therapy and regeneration. This approach, also frequently used by regional scientists, increases the progressive need for cooperation between the Academia and industry. One line of research is devoted to the design of surfaces and model predictions of attachment of growing cells such as osteoblasts on TiO_2_ nanotubes, as presented by Aleš Iglič from the University of Ljubljana (5), or to tissue engineering by combining cells, biomaterial, and environmental cues to design autologous bone tissue, which was presented as an enterprise goal of the Ljubljana-based company Educell (presentation by Lenart Girandon).

## Conclusion

RBC series is a reliable and sustainable means of networking for scientists from the field of biophysics in the region. The conference demonstrated that medical applications of biophysics in the region were widespread and justify ongoing research. The multidisciplinary topics covered a wide field from instrumental methods for therapy and diagnostics to tissue engineering for regeneration. The RBC-established concept of trans-border collaboration in the region is a solid base for further steps in this direction.
